# Is lipoprotein(a) measurement important for cardiovascular risk stratification in children and adolescents?

**DOI:** 10.1186/s13052-024-01732-8

**Published:** 2024-09-04

**Authors:** Marco Giussani, Antonina Orlando, Elena Tassistro, Erminio Torresani, Giulia Lieti, Ilenia Patti, Claudia Colombrita, Ilaria Bulgarelli, Laura Antolini, Gianfranco Parati, Simonetta Genovesi

**Affiliations:** 1 Istituto Auxologico Italiano, IRCCS, Via L. Ariosto 13, Milano, 20145 Italy; 2 Biostatistics and Clinical Epidemiology, Fondazione IRCCS San Gerardo dei Tintori, Monza, Italy; 3 School of Medicine and Surgery, University of Milano-Bicocca, Monza, Italy; 4 Bicocca Center of Bioinformatics, Biostatistics and Bioimaging (B4 Center), University of Milano-Bicocca, Monza, Italy

**Keywords:** Lipoprotein (a), LDL cholesterol, Children, Cardiovascular risk

## Abstract

**Background:**

Elevated lipoprotein (Lp(a)) levels are associated with increased risk of atherosclerotic processes and cardiovascular events in adults. The amount of Lp(a) is mainly genetically determined. Therefore, it is important to identify individuals with elevated Lp(a) as early as possible, particularly if other cardiovascular risk factors are present. The purpose of the study was to investigate whether, in a population of children and adolescents already followed for the presence of one or more cardiovascular risk factors (elevated blood pressure (BP), and/or excess body weight, and/or dyslipidemia), the measurement of Lp(a) can be useful for better stratifying their risk profile.

**Methods:**

In a sample of 195 children and adolescents, height, body weight, waist circumference and systolic (SBP) and diastolic (DBP) BP were measured. Body Mass Index (BMI) and SBP and DBP z-scores were calculated. Plasma Lp(a), total cholesterol, high-density lipoprotein (HDL), triglycerides, glucose, insulin, uric acid and creatinine were assessed. Low-density lipoprotein (LDL) cholesterol was calculated with the Friedewald formula. High Lp(a) was defined as *≥* 75 nmol/L and high LDL cholesterol as *≥* 3.37 mmol/L.

**Results:**

Our sample of children and adolescents (54.4% males, mean age 11.5 years) had median LDL cholesterol and Lp(a) values equal to 2.54 (interquartile range, IQR: 2.07–3.06) mmol/L and 22 (IQR: 7.8–68.6) nmol/L respectively. 13.8% of children had LDL cholesterol *≥* 3.37 mmol/L and 22.6 Lp(a) values *≥* 75 nmol/L. Lp(a) values were higher in children of normal weight than in those with excess weight (*p* = 0.007), but the difference disappeared if normal weight children referred for dyslipidemia only were excluded from the analysis (*p* = 0.210). 69.4% of children had normal Lp(a) and LDL cholesterol values and only 6.2% showed both elevated Lp(a) and LDL cholesterol levels. However, 16.6% of the sample, despite having normal LDL cholesterol, had elevated Lp(a) values. Multivariable analyses showed a significant association of LDL cholesterol both with Lp(a) values, and with the presence of elevated Lp(a) levels. For each mmol/L increase in LDL cholesterol the risk of having an elevated Lp(a) value increased by 73%. There was an inverse correlation between BMI z-score and Lp(a). Neither BP z-scores, nor other biochemical parameters were associated with Lp(a).

**Conclusions:**

In our population more than one out of five children had elevated Lp(a) values, and in about 17% of children elevated Lp(a) values were present in the absence of increased LDL cholesterol. Our results suggest that Lp(a) measurement can be useful to better define the cardiovascular risk profile in children and adolescents already followed for the presence of other cardiovascular risk factors such as elevated BP, excess body weight and high LDL cholesterol.

**Supplementary Information:**

The online version contains supplementary material available at 10.1186/s13052-024-01732-8.

## Introduction

Small-type lipoprotein (Lp(a)) consists of a lipoprotein-like-LDL joined to a glycoprotein, called apolipoprotein(a) [apo(a)], by a single disulfide bridge with apo B100. Lp(a) is expressed by the LPA gene whose two alleles encode apo(a) that can be different from each other. The amount of Lp(a) in adults is constant and is 80% genetically determined [1–3]. In newborns, Lp(a) values are very low, but increase rapidly during the first few months of life until they reach adult-like values at two years of age. According to some authors, modest further increases may occur [4–6]. There is a great deal of evidence that elevated Lp(a) levels are associated with increased atherosclerotic processes, cardiovascular events, and calcific aortic valve stenosis in adulthood [7–11]. The pro-atherosclerotic action of Lp(a) involves several mechanisms: its cholesterol content, its ability to bind to structures in the vascular wall and stimulate chemotaxis, and the pro-inflammatory activity of oxidized phospholipids present in the like-LDL and apo(a). Finally, pro-thrombotic activity has been suggested [12–18]. Lp(a) values have an in-continuum relationship with cardiovascular risk, so a precise cut-off defining increased risk is not established; however, there is agreement that values exceeding 75 nmol/L can be considered elevated [19, 20]. In light of these considerations, it might be useful to identify individuals with elevated Lp(a) levels as early as possible, particularly if other cardiovascular risk factors are present, warranting increased attention for the implementation of preventive interventions.

The purpose of this study was to investigate whether, in a population of children and adolescents already followed for the presence of one or more cardiovascular risk factors (i.e. elevated blood pressure (BP), and/or excess body weight, and/or dyslipidemia), the measurement of Lp(a) can be useful for better stratifying their risk profile.

## Methods

### Subjects

In this prospective cross-sectional study, we followed 195 consecutive children and adolescents, referred by their family paediatricians (from 07/05/2019 to 11/04/2023) to the Cardiovascular Risk Assessment in Children Unit, Istituto Auxologico, Milan, for the presence of at least one of the following cardiovascular risk factors: excess body weight, elevated blood pressure, dyslipidemia. The exclusion criteria were: presence of Type 1 or 2 diabetes mellitus, secondary arterial hypertension, ongoing pharmacological treatment. The achieved sample size enables to estimate a 95% confidence interval (CI) for the probability of having a Lp(a) value ≥ 75 nmol/L with a maximum length of 15% points using the Clopper-Pearson exact interval in the case of maximum variability where the point estimate will result equal to 50%.

### Measurement of anthropometric variables and blood pressure

The anthropometric parameters measured were height, body weight, and waist circumference. The precision applied for data recording was 100 g for body weight (SECA robusta 813, Hamburg Germany) and 1 mm for height (altimeter SECA 222, Hamburg Germany). Body mass index was calculated as participants’ weight in kilograms divided by the square of height in meters. Body mass index z-scores were calculated using prevention tables from the Centers for Disease and Control (CDC) prevention charts available at https://www.cdc.gov/growthcharts/clinical_charts.htm (accessed on 31 march 2024). Weight class was defined according to the International Obesity Task Force classification [21] distinguishing between normal weight, overweight and obese individuals. Waist circumference was recorded with an accuracy of 0.5 cm while the study participants were standing. Waist measurement was performed as recommended by Lohman et al. [22]. Waist-to-height ratio was obtained by dividing waist circumference by height. Blood pressure was measured with an oscillometric device validated for use in children and recommended by the 2016 European Society of Hypertension Pediatric guidelines (Omron 705IT; Omron Co, Kyoto, Japan); special care was taken to use an appropriately sized cuff. Blood pressure values were recorded after a rest period of at least 5 min and while participants were seated. The measurement was taken 3 times (at intervals of a few minutes), and the mean value of the second and third measurements was recorded. The percentiles and z-scores of systolic (SBP) and diastolic (DBP) BP were calculated based on nomograms from the National High Blood Pressure Education Program (NHBPEP) Working Group on High Blood Pressure in Children and Adolescents [23, 24]. Children were classified according to the mean of the two measurements as follows: normotensive (NT) if the percentiles of SBP and DBP were both < 90th; high normal (HN) if the percentiles of SBP and/or DBP were ≥ 90th, but both < 95th; hypertensive (HT) if the percentiles of SBP and/or DBP were ≥ 95th.

### Biochemical parameters

The venous sampling was performed after at least 12 h of fasting and the assays of all parameters were performed on the same day as the venous sampling, except for Lp(a) whose assay was carried out in a single time on all serum samples stored frozen at -80 °C. For the measurement of total cholesterol, HDL, triglycerides, glucose, and insulin, commercial kits were used (Cobas Roche colorimetric enzymatic cholesterol Gen.2 test, for total cholesterol assay; homogeneous-phase colorimetric enzymatic test HDL cholesterol Gen.4 Cobas Roche, for HDL cholesterol; colorimetric enzymatic test Triglycerides Cobas Roche, for triglyceride assay; hexokinase enzymatic method Glucose HK Gen.3 Cobas Roche, for glucose assay; immunoassay in ElectroChemiLuminescence Elecsys Insulin Cobas Roche, for insulin assay; colorimetric enzymatic test Uric Acid 2 Cobas Roche for uric acid assay).

LDL cholesterol was calculated with the Friedewald formula (LDL cholesterol = Total cholesterol – HDL cholesterol – 1/5 triglycerides). The Homeostasis Model Assessment (HOMA)-index was calculated by dividing the product of serum insulin (µU/mL) and serum glucose (mmol/L) by 22.5 [25]. Glomerular filtration rate was estimated (eGFR) by the simplified Schwartz formula [26]. The Lp(a) assay was performed with an enhanced particle immunoturbidimetric method using Roche/Hitachi Cobas c systems. The principle of the method is as follows: human lipoprotein (a) agglutinates latex particles coated with anti‑Lp(a) antibodies. The precipitate is determined turbidimetrically at 800/660 nm. Results were expressed in nmol/L. High Lp(a) was defined as ≥ 75 nmol/L [20] and high LDL cholesterol as *≥* 3.37 mmol/L (corresponding to ≥ 130 mg/dL) [27].

### Statistical analysis

The characteristics of the sample were described by mean and standard deviation (SD) or median and interquartile range (IQR), as appropriate according to normal distribution, if the variables were continuous and by frequencies and percentages if they were categorical.

The univariate association between Lp(a) and HDL cholesterol was represented in a scatterplot, where the Pearson correlation was calculated and the p-value obtained from the correlation test was displayed. The distribution of Lp(a) values in weight classes was represented in boxplots. A bivariate linear regression model was performed and the obtained p-value was displayed.

Univariate analyses to compare the characteristics of the cohort in the four phenotypes (Low Lp(a)/Low LDL cholesterol, High Lp(a)/Low LDL cholesterol, Low Lp(a)/High LDL cholesterol, High Lp(a)/High LDL cholesterol) were conducted using the Mann − Whitney or the t- test (as appropriate) in case of continuous variables, and through the Chi-Square test in case of categorical variables with α = 0.05 significance level. Post hoc pairwise comparisons were obtained according to the Bonferroni correction significance level (α/6).

Multivariable linear and logistic regression models were used to assess the impact of sex, age, parental dyslipidemia, BMI z-score (or as an alternative waist-to-height ratio) and LDL cholesterol on continuous and elevated Lp(a) values respectively, with p-values < 0.05 considered to be statistically significant.

Statistical analyses were performed with R 4.3.1 (http://www.R-project.org). All p-values were 2-sided.

## Results

One sample of 195 children and adolescents was included in the study (54.4% males, mean age 11.5 *±* 2.7 years).

The main anthropometric and clinical characteristics of the population are shown in Table 1. 35% of the parents had a history of dyslipidemia and 5.7% of early cardiovascular disease (before age 55). 78% of the sample was in excess weight and 63% had waist-to-height ratio > 50%. Approximately 20% of the children had SBP and/or DBP values *≥* 90th percentile.

**Table 1:** Anthropometric and clinical characteristics of the study population

	Overall (*N* = 195)
Males, n (%)	106 (54.4)
Age (years), mean (SD)	11.5 (2.7)
Birth weight (kg), median (IQR)	3.300(2.90–3.600)
Early cardiovascular disease in parents, n (%)	11 (5.7)
Parental dyslipidemia, n (%)	68 (34.9)
Smoking in cohabitants, n (%)	162 (84.4)
Weight (kg), median (IQR)	56.1 (42.9–67.6)
Height (cm), median (IQR)	150.0 (138.0-159.0)
Waist circumference (cm), median (IQR)	78.0 (70.3–85.3)
BMI, mean (SD)	24.7 (5.1)
BMI z-score, median (IQR)	1.8 (1.1–2.1)
Weight class, n (%)	
* Normal weight*	43 (22.1)
* Overweight*	54 (27.7)
* Obese*	98 (50.3)
Waist-to-height ratio, median (IQR)	52.9 (47.5–57.4)
Waist-to-height ratio > 50%, n (%)	123 (63.1)
Systolic BP (mmHg), median (IQR)	110.0 (103.0-119.5)
Systolic BP z-score, median (IQR)	0.48 (-0.16-1.08)
Diastolic BP (mmHg), median (IQR)	64.0 (59.5–70.0)
Diastolic BP z-score, median (IQR)	0.15 (-0.21-0.64)
BP category, n (%)	
* Normotension*	155 (79.4)
* High-normal*	20 (10.3)
* Hypertension*	20 (10.3)
Cholesterol (mmol/L:), median (IQR)	4.35 (3.73–4.97)
HDL cholesterol (mmol/L), median (IQR)	1.29 (1.11–1.53)
LDL cholesterol (mmol/L), median (IQR)	2.54 (2.07–3.06)
LDL cholesterol ≥ 3.37 mmol/L n (%)	27 (13.8)
Triglycerides (mmol/L), median (IQR)	0.88 (0.66–1.18)
Glucose (mmol/L), mean (SD)	4.77 (0.32)
Insulin (µU/mL), median (IQR)	13.8 (9.9–21.0)
HOMA index*, median (IQR)	2.9 (2.1–4.4)
Uric acid (µmol/L), median (IQR)	279.6 (237.9-322.7)
Creatinine (µmol/L), median (IQR)	48.62 (42.43–57.46)
eGFR (ml/min/1.73m^2^), median (IQR)	145.4 (129.0-163.0)
C-Reactive protein (mg/L), median (IQR)	1.0 (0.8-3.0)
Lp(a) (nmol/L), median (IQR)	22.0 (7.8–68.6)
Lp(a) ≥ 75 nmol/L, n (%)	44 (22.6)

SD = Standard Deviation, IQR = interquartile range; BMI = Body Mass Index; BP = Blood Pressure; eGFR = estimated Glomerular Filtration Rate; Lip(a) = lipoprotein (a)

* Calculated as plasma insulin (µU/mL) * plasma glucose (mmol/L) / 22.5

Median LDL cholesterol and Lp(a) values were 2.54 (IQR: 2.07–3.06) mmol/L and 22 (IQR: 7.8–68.6) nmol/L respectively. 14% of children had LDL cholesterol *≥* 3.37 mmol/L and 22.6% (95% CI: 16.9–29.2%) had Lp(a) values *≥* 75 nmol/L (Table 1).

**Fig. 1:**
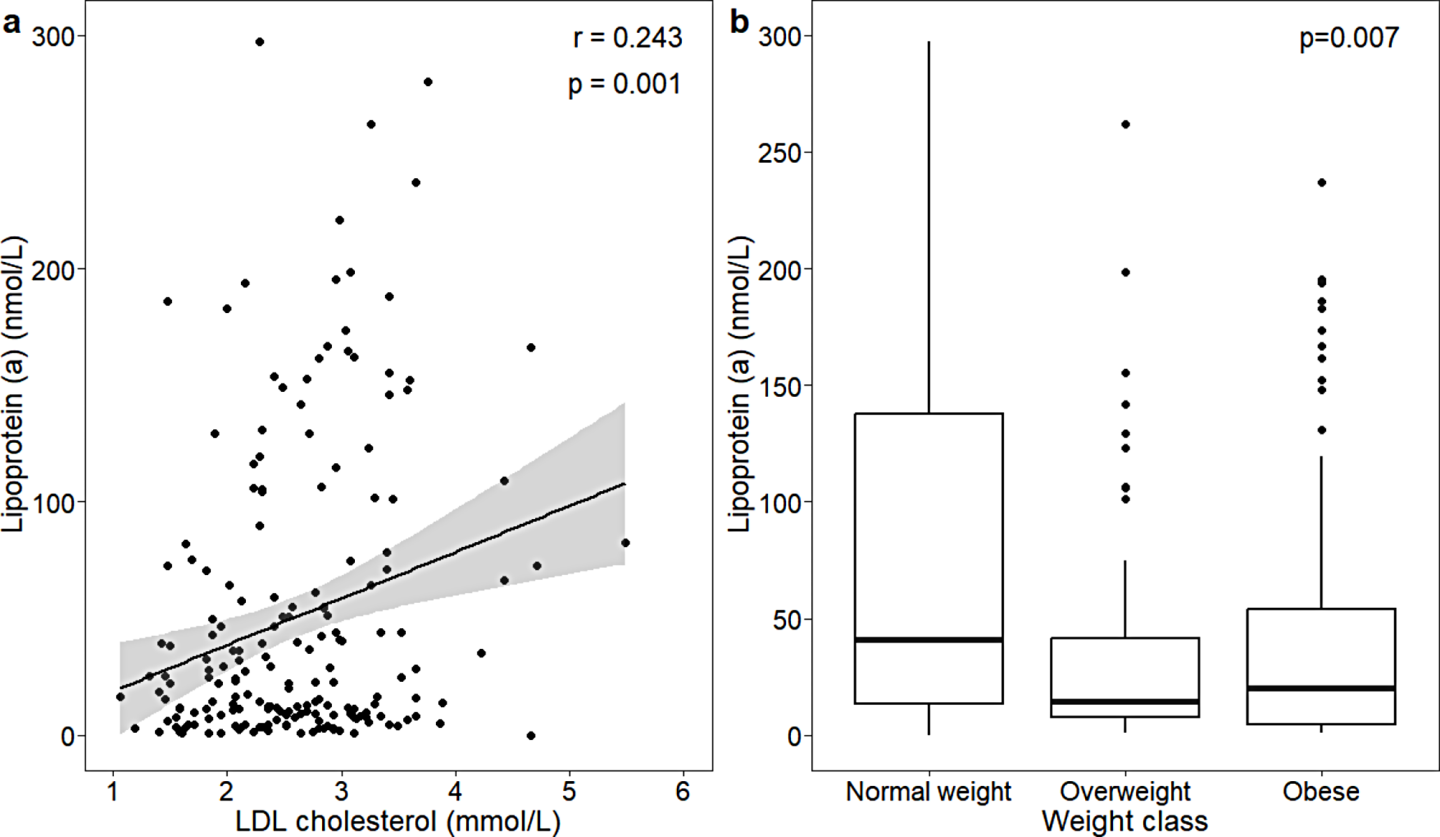
Relationship between plasma LDL cholesterol and lipoprotein (**a**) and between weight classes and lipoprotein values (**b**). Normal Weight vs. Overweight *p* = 0.011; Normal Weight vs. Obese *p* = 0.012

There was a significant positive correlation between LDL cholesterol values and Lp(a) values (*r* = 0.243, *p* = 0.001) and an inverse association between weight class and Lp(a) values (median, IQR: 40.7 (13.2-137.6) nmol/L for normal weight, 14.4 (7.7–41.6) nmol/L for overweight, 19.9 (4.8–53.7) nmol/L for obese, *p* = 0.007) (Fig. 1). This latter relationship was lost if the 26 normal weight children referred by pediatricians for the presence of dyslipidemia only were excluded from the analysis (median, IQR: 24.3 (12.7-122.7) nmol/L for normal weight, 13.9 (7.7–42.5) nmol/L for overweight, 19.9 (4.8–53.7) nmol/L for obese, *p* = 0.21, data not shown). Total cholesterol was positively correlated with Lp(a) values (*r* = 0.259, *p* < 0.001, Supplementary Figure S1) and parental family history of dyslipidemia was weakly associated with higher Lp(a) values (median, IQR: 37.6 (8.7–87.1 nmol/L) for presence of parental dyslipidemia, 16.4 (6.6–53.0) nmol/L for no parental dyslipidemia, *p* = 0.04, Supplementary Figure S1).

Table 2 shows the anthropometric and clinical characteristics of the four phenotypes (Low Lp(a)/Low LDL cholesterol, High Lp(a)/Low LDL cholesterol, Low Lp(a)/High LDL cholesterol, High Lp(a)/High LDL cholesterol) detected in our sample, from which we excluded 2 subjects without LDL cholesterol measurement. Approximately 70% of the children had normal Lp(a) and LDL cholesterol values and only 6% showed both elevated Lp(a) and LDL cholesterol levels. However, almost 17% of the sample, despite having normal LDL cholesterol values, had elevated Lp(a) values. As expected, the total cholesterol value was higher in the two subgroups with high LDL cholesterol levels (*p* < 0.001). The highest prevalence of parental dyslipidemia was in the Low Lp(a)/High LDL cholesterol subgroup and the lowest in the Low Lp(a)/Low LDL cholesterol subgroup (*p* = 0.001, overall test). Despite no statistically significant differences were highlighted in post-hoc pairwise comparisons, highest BMI z-score and HOMA-index values were detected in children with low Lp(a) and low LDL cholesterol values, whereas the lowest values of BMI z-score and HOMA-index were measured in children with both high Lp(a) and LDL cholesterol values (global *p* < 0.05 for both variables).

**Table 2:** Anthropometric and clinical characteristics of the four phenotypes (Low Lipoprotein (a)/Low LDL cholesterol, high lipoprotein (a)/Low LDL cholesterol, Low Lipoprotein (a)/High LDL cholesterol, high lipoprotein (a)/High LDL cholesterol) detected in our study population

	Low Lp(a)/low LDL (*N* = 134, 69.4%)	High Lp(a)/low LDL (*N* = 32, 16.6%)	Low Lp(a)/high LDL (*N* = 15, 7.8%)	High Lp(a)/high LDL (*N* = 12, 6.2%)	*P*
Males, n (%)	64 (47.8)	20 (62.5)	11 (73.3)	10 (83.3)	0.024
Age (years), mean (SD)	11.6 (2.7)	12.3 (2.5)	10.8 (2.6)	10.4 (3.4)	0.128
Birth weight (kg), median (IQR)	3.3 (2.9–3.6)	3.3 (2.9–3.5)	3.5 (3.1–3.9)	3.4 (3.2–3.5)	0.457
Early cardiovascular disease in parents, n (%)	7 (5.3)	2 (6.2)	2 (13.3)	0 (0.0)	0.501
Parental dyslipidemia, n (%)	36 (26.9)	13 (40.6)	11 (73.3) ^#^	7 (58.3)	0.001
Smoking in cohabitants, n (%)	110 (84.0)	28 (87.5)	13 (86.7)	10 (83.3)	0.958
BMI z-score, median (IQR)	1.9 (1.4–2.2)	1.6 (0.6–2.9)	1.6 (0.3–2.2)	1.1 (-1.0-1.8)	0.020
Weight class, n (%)					0.010
* Normal weight*	19 (14.2)	11 (34.4)	6 (40.0)	6 (50.0) ^#^	
* Overweight*	43 (32.1)	7 (21.9)	2 (13.3)	2 (16.7)	
* Obese*	72 (53.7)	14 (43.8)	7 (46.7)	4 (33.3)	
Waist-to-height ratio, median (IQR)	53.6 (49.1–57.7)	49.7 (44.2–54.5)^#^	55.6 (45.0-60.8)	48.6 (44.8–52.5)	0.015
Waist-to-height ratio > 50%, n (%)	92 (68.7)	16 (50.0)	9 (60.0)	5 (41.7)	0.087
Systolic BP (mmHg), median (IQR)	110 (103–119)	112.0 (107–121)	102(96–111)	110 (96–120)	0.076
Systolic BP z-score, median (IQR)	0.57 (-0.07-1.08)	0.50 (-0.16-1.08)	-0.13 (-0.48-0.85)	0.27 (-0.49-0.71)	0.190
Diastolic BP (mmHg), median (IQR)	64 (60–71)	66 (63–71)	59 (54–67)	64 (59–68)	0.282
Diastolic BP z-score, median (IQR)	0.14 (-0.27- 0.64)	0.22 (-0.03-0.66)	-0.07 (-0.46-0.43)	0.16 (-0.06-0.57)	0.637
BP category, n (%)					0.970
* Normotension*	104 (77.6)	27 (84.4)	12 (80.0)	10 (83.4)	
* High-normal*	15 (11.2)	2 (6.2)	2 (13.3)	1 (8.3)	
* Hypertension*	15 (11.2)	3 (9.4)	1 (6.7)	1 (8.3)	
Cholesterol (mmol/L), median (IQR)	4.04 (3.56–4.68)	4.47 (3.98–4.90)^#^	5.75 (5.24–6.33)^#$^	5.43 (5.28–5.98)^#$^	< 0.001
HDL cholesterol (mmol/L), median (IQR)	1.28 (1.09–1.50)	1.31 (1.18–1.59)	1.37 (1.06–1.61)	1.45 (1.26–1.53)	0.420
Triglycerides (mmol/L), median (IQR)	0.89 (0.66–1.14)	0.87 (0.64–1.22)	1.08 (0.85–1.36)	0.76 (0.60–0.92)	0.125
Glucose (mmol/L), mean (SD)	4.79 (0.33)	4.77 (0.20)	4.70 (0.31)	4.64 (0.44)	0.351
Insulin (µmol/L), median (IQR)	15.4 (10.6–21.1)	13.5 (9.9–21.5)	11.8 (7.7–14.8)	8.9 (3.9–15.9)	0.042
HOMA index*, median (IQR)	3.2 (2.2–4.4)	2.9 (2.1–4.7)	2.5 (1.5–3.1)	2.0 (0.8–3.4)	0.040
Uric acid (µmol/L), median (IQR)	273.6 (237.9-315.2)	279.6 (237.9-333.1)	237.9 (199.3-306.3)	303.4 (237.9-352.4)	0.576
Creatinine (µmol/L), median (IQR)	50.39 (44.20-58.34)	45.97 (43.32–58.34)	45.97 (40.66–54.81)	47.74 (36.24–56.58)	0.589
eGFR (ml/min/1.73m^2^), median (IQR)	144.0 (129.0-160.8)	149.2 (132.8-165.3)	148.0 (133.0-157.5)	154.0 (130.7-164.5)	0.812
C-Reactive protein (mg/L), median (IQR)	1.0 (1.0–3.0)	1.0 (0.00–3.0)	1.0 (0.00-1.8)	2.0 (1.0-4.5)	0.218

SD = Standard Deviation, IQR = interquartile range; Lp(a) = lipoprotein (a); BMI = Body Mass Index; BP = Blood Pressure; eGFR = estimated Glomerular Filtration Rate

* Calculated as plasma insulin (µU/mL) * plasma glucose (mmol/L) / 22.5

Post hoc pairwise comparisons p-values < α/6: ^#^, versus Low Lp(a)/Low LDL; ^$^, Low Lp(a)/High LDL or High Lp(a)/High LDL versus High Lp(a)/Low LDL

Multivariable analyses confirmed a significant association between LDL cholesterol values both with Lp(a) values considered as a continuous variable (Table 3, Model 1), and with the presence of elevated Lp(a) values (i.e. *≥*75nmol/L) (Table 3, Model 2). For each mmol/L increase in LDL cholesterol the risk of having a pathological Lp(a) value increased by 73%. In multivariable models, the inverse relationship between BMI z-score and Lp(a) was confirmed (Table 3). The results were comparable when the BMI z-score was replaced by the Waist-to-height ratio (Supplementary Table S1).

**Table 3:** Effect of sex, age, parental dyslipidemia, BMI z-score and LDL cholesterol on continuous (model 1) and elevated (≥ 75 nmol/L, model 2) lipoprotein (a) values by multiple linear and logistic models respectively

	Model 1			Model 2		
Variable	b	(95% CI)	*P*	OR	(95% CI)	*P*
Intercept	-5.31	(-62.30; 51.67)	0.854	-	-	-
Sex (males vs. females)	12.18	(-5.17; 29.52)	0.168	1.90	(0.89; 4.18)	0.101
Age (years)	2.32	(-0.98; 5.61)	0.167	1.04	(0.91; 1.20)	0.554
Parental dyslipidemia	1.25	(-18.61; 21.12)	0.901	1.02	(0.44; 2.28)	0.963
BMI z-score	-13.30	(-21.80; -4.80)	0.002	0.65	(0.47; 0.90)	0.011
LDL cholesterol (mmol/L)	15.94	(3.25; 28.62)	0.014	1.73	(1.03; 2.98)	0.042

b = multivariable coefficient; CI = confidence interval; OR = Odds Ratio; P = p-value; BMI, body mass index;

LDL, Low-density lipoprotein

## Discussion

In a sample of children and adolescents already known to be at cardiovascular risk (presence of excess body weight and/or elevated BP values and/or dyslipidemia) the prevalence of high Lp(a) values was high (22.6%). Lp(a) values were associated with LDL cholesterol levels, however a significant percentage of children (17%) had high Lp(a) values despite not having an increase in LDL cholesterol.

A relevant data from the study is the high number of children with high levels of Lp(a): one child out of every 5 had elevated values (*≥* 75 nmol/L) of Lp(a). This result is particularly relevant, as ours is already a population at cardiovascular risk due to other factors. The prevalence of elevated Lp(a) levels in our sample is similar to that found in adults in the general European population [28]. The plasma value of Lp(a) depends on the hepatic production of apo(a), encoded by the LPA gene [29, 30], independently of the level of LDL cholesterol. Thus, some people may have high Lp(a) and low LDL cholesterol, low Lp(a) and high LDL cholesterol, or a combination of the two patterns. It is important to note that in our study we found elevated Lp(a) values in a number of children (about 17%, phenotype High Lp(a)/Low LDL) who had not been referred to our clinic for the presence of dyslipidemia. These children, in the absence of Lp(a) measurement, would have been considered as having a normal lipid profile.

An increase in Lp(a) has been described in the presence of renal failure and inflammatory diseases [31, 32]. However, this should not be the case in our population, because the eGFR of the children included in this study was within normal limits and no child had increased C-reactive protein values.

In our sample, children with at least one parent with dyslipidemia had higher Lp(a) values, however this relationship was not confirmed in the multivariable statistical model. Moreover, we did not detect an increase in early cardiovascular disease (cardiovascular events before age 55) in parents of children with higher Lp(a) levels. It should be underlined, however, that the average age of our sample was around 11 years and the majority of parents were under 55 years old, therefore the cumulative incidence might be “falsely low”, because of a short duration of observation. A correlation between Lp(a) values in children and parents was previously described [6, 33]. Unfortunately, the latter data was not available in our study, and we could not investigate this issue.

A very high prevalence of smoking cohabitants (> 80%) was reported in our sample. The deleterious effect of active, but also passive smoking [34] is well known. The elevated prevalence of smoking exposure suggests that our population came from “cardiovascular unhealthy” families. On the other hand, our population was known to be at cardiovascular risk, and the high prevalence of smokers (parents, older siblings, grandparents or other cohabitants) in the family represents an additional risk factor for these children. Although there is no association between Lip (a) levels and smoking, a more accurate stratification of cardiovascular risk obtained by measuring Lp(a), could be useful in alerting parents to eliminate all modifiable risk factors from the lifes of their children, including passive smoking.

Approximately 14% of our population had a pathological LDL cholesterol value (*≥* 3.37 mmol/L). As already described [35, 36] parental dyslipidemia was more prevalent in the two subgroups of children with high LDL cholesterol values, independently of those of Lp(a). However, given that Lp(a) contributes to the value of LDL cholesterol, these two phenotypes could be the expression of different clinical situations. A child with low Lp(a) and high LDL cholesterol is more suspected, particularly if there is a history of parental dyslipidemia, of having heterozygous familial hypercholesterolemia. On the contrary, with the same LDL values, the presence of high Lp(a) values could make this diagnosis less likely [37, 38].

We found a significant correlation between Lp(a) values and those of total cholesterol and LDL cholesterol. This data is not unexpected and can be explained by the fact that the cholesterol content of the LDL-like portion of Lp(a) is measured together with total cholesterol and LDL cholesterol. This amount ranges from 30 to 40% of total LDL [39]. Unexpectedly, we found an inverse association between BMI z-score (or Waist-to-height ratio) and Lp(a) values. One explanation for this finding may be that several children were referred to our clinic only because they had dyslipidemia, despite being normal weight or even low weight, and with normal BP and HOMA-index values. As described in Fig. 1, the inverse association between BMI and Lp(a) values was no longer significant if individuals referred only for the presence of elevated LDL-cholesterol values were removed from the analysis.

We did not find correlations with high Lp(a) values and other blood chemistry parameters. Moreover, birth weight was not correlated with Lp(a) values in our population. In our sample we did not find differences in Lp(a) values associated with age, a result that is in disagreement with what was reported by a group of Dutch researchers who, by analyzing a large population of children with dyslipidemia, found that older individuals had higher Lp(a) values [4]. Several authors have reported an increase in Lp(a) levels between the first months of life and a stabilization of values at two years [6]. On this point our study provides no information because all the included children were older than two years of age.

### Limitations of the study

The results of our study cannot be generalized to a general pediatric population, as ours is a sample with increased cardiovascular risk, probably coming from an unhealthy family background as demonstrated by the high prevalence of families with smokers. However, our study had no epidemiological purpose. We wanted to assess whether the Lp(a) measurement could help to better stratify cardiovascular risk in a sample of children from a known at-risk population. Moreover, the sample was relatively small. Another limitation is the lack of data relating to parents’ Lp(a) values.

## Conclusions

The possible clinical implications of our study are important. The amount of Lp(a) in adults is constant and is determined 80% by genetic factors. It has been shown that high Lp(a) values in pediatric age are maintained throughout life [3]. On the background of the acknowledged adverse prognostic role of high Lp(a), it is reasonable to assume that a child with high Lp(a) values is at greater risk of experiencing a cardiovascular event over the course of future life than one with low Lp(a) values. However, only longitudinal studies can tell us what the burden of having an elevated Lp(a) finding from pediatric age will be in relation to future cardiovascular events. For this reason, Lp(a) measurement may be useful to better define the cardiovascular risk profile in children and adolescents already followed due to the presence of other cardiovascular risk factors such as arterial hypertension, excess body weight and high LDL cholesterol values. The finding of high Lp(a) levels can represent an additional concern for the health of these children and a more aggressive intervention on modifiable risk factors and closer clinical follow-up are important, in the absence of a specific treatment aimed at reducing Lp(a) values. The hope is that in the near future a specific therapy may become available at least for individuals with the highest Lp(a) values.

## Electronic supplementary material

Below is the link to the electronic supplementary material.

Supplementary Material 1

## Data Availability

The data presented in this study are available upon reasonable request from the corresponding author.

## Abbreviations

Lp(a): Lipoprotein a
BP: Blood pressure
SBP: Systolic blood pressure
DBP: Systolic blood pressure
BMI: Body Mass Index
HOMA: Homeostasis Model Assessment
HDL: High-density lipoprotein
LDL: Low-density lipoprotein
IQR: Interquartile range
apo(a): Apolipoprotein(a)
NT: Normotensive
HN: High normal
HT: Hypertensive
eGFR: Estimated glomerular filtration
CRP: C-reactive protein
