# Supplementary material for: Is lipoprotein(a) measurement important for cardiovascular risk stratification in children and adolescents?
Source: Ital J Pediatr. 2024 Sep 4;50:161. doi: 10.1186/s13052-024-01732-8 (PMC11373248; doi:10.1186/s13052-024-01732-8)
Supplement: Supplementary file 1 — Supplementary Material 1 [file 13052_2024_1732_MOESM1_ESM.docx]

**Figure S1**: Relationship between plasma total cholesterol and lipoprotein (a) values and parental history of dyslipidemia (b) and lipoprotein (a) levels.

**
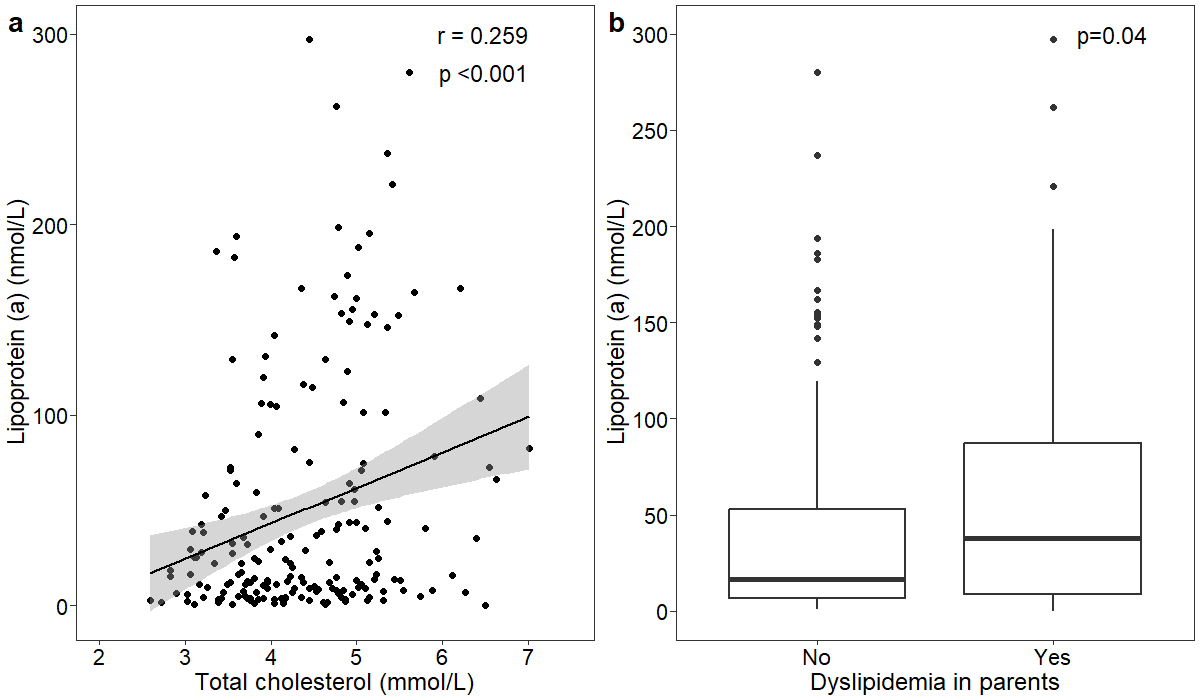
**

**Table S1.** Effect of sex, age parental history of dyslipidemia, Waist-to-Height ratio and LDL cholesterol on continuous (Model 3) and elevated (Model 4) Lipoprotein (a) values (≥75 nmol/L) by multiple linear and logistic models, respectively.

|  | **Model 3** | | | **Model 4** | | |
| --- | --- | --- | --- | --- | --- | --- |
| *Variable* | *b* | *(95% CI)* | *P* | *OR* | *(95% CI)* | *P* |
| Intercept | 68.42 | (-17.15, 153.99) | 0.116 | - | - | - |
| Sex (males vs females) | 11.87 | (-5.44, 29.19) | 0.178 | 1.89 | (0.89, 4.13) | 0.104 |
| Age (years) | 2.15 | (-1.15, 5.46) | 0.201 | 1.03 | (0.90, 1.19) | 0.652 |
| Parental history of dyslipidemia | -0.06 | (-20.03, 19.90) | 0.995 | 0.95 | (0.41, 2.13) | 0.895 |
| Waist-to-Height ratio | -1.81 | (-2.94, -0.68) | 0.002 | 0.93 | (0.89, 0.98) | 0.007 |
| LDL cholesterol (mmol/L) | 17.72 | (5.17, 30.27) | 0.006 | 1.84 | (1.09, 3.17) | 0.024 |

b = multivariable coefficient; CI = confidence interval; OR = Odds Ratio; P = p-value; LDL, Low-density lipoprotein
